# Supplementary material for: Curated and harmonised transcriptomics datasets of interstitial lung diseases
Source: Data Brief. 2025 Oct 14;63:112139. doi: 10.1016/j.dib.2025.112139 (PMC12581653; doi:10.1016/j.dib.2025.112139)
Supplement: Supplementary file 1 [file mmc1.zip › Supplementary_material/RNA-seq/fastQC reports/GSE180415/SRR15194701_1_fastqc.html]

SRR15194701\_1.fastq.gz FastQC Report 

FastQC Report

Wed 25 Oct 2023  
SRR15194701\_1.fastq.gz

## Summary

- Basic Statistics
- Per base sequence quality
- Per sequence quality scores
- Per base sequence content
- Per sequence GC content
- Per base N content
- Sequence Length Distribution
- Sequence Duplication Levels
- Overrepresented sequences
- Adapter Content

## Basic Statistics

| Measure | Value |
| --- | --- |
| Filename | SRR15194701\_1.fastq.gz |
| File type | Conventional base calls |
| Encoding | Sanger / Illumina 1.9 |
| Total Sequences | 38790849 |
| Sequences flagged as poor quality | 0 |
| Sequence length | 35-80 |
| %GC | 54 |

## Per base sequence quality

## Per sequence quality scores

## Per base sequence content

## Per sequence GC content

## Per base N content

## Sequence Length Distribution

## Sequence Duplication Levels

## Overrepresented sequences

| Sequence | Count | Percentage | Possible Source |
| --- | --- | --- | --- |
| CTCAGACCGCGTTCTCTCCCTCTCACTCCCCAATACGGAGAGAAGAACGA | 104164 | 0.2685272498160584 | No Hit |
| CCAGGCTGGAGTGCAGTGGCTATTCACAGGCGCGATCCCACTACTGATCA | 102093 | 0.2631883617705815 | No Hit |
| GCTCAGGCTGGAGTGCAGTGGCTATTCACAGGCGCGATCCCACTACTGAT | 62259 | 0.16049919402382765 | No Hit |
| CAGGCTGGAGTGCAGTGGCTATTCACAGGCGCGATCCCACTACTGATCAG | 57719 | 0.14879540275078795 | No Hit |
| GGCTGGAGTGCAGTGGCTATTCACAGGCGCGATCCCACTACTGATCAGCA | 57577 | 0.1484293370325563 | No Hit |
| GGGCGGGCTCCCGGCCCCGGCCGACGCGCCGCGAGGCGAGCCGGGCGGGC | 49190 | 0.12680825830855108 | No Hit |
| GTGGCTATTCACAGGCGCGATCCCACTACTGATCAGCACGGGAGTTTTGA | 48788 | 0.1257719314160925 | No Hit |
| CTGGAGTCTTGGAAGCTTGACTACCCTACGTTCTCCTACAAATGGACCTT | 42577 | 0.10976042313484813 | No Hit |
| GCCCAGGCTGGAGTGCAGTGGCTATTCACAGGCGCGATCCCACTACTGAT | 41421 | 0.10678033883713141 | No Hit |
| CTCCGTTTCCGACCTGGGCCGGTTCACCCCTCCTTAGGCAACCTGGTGGT | 40524 | 0.10446793778604846 | No Hit |

## Adapter Content

Produced by FastQC (version 0.11.7)
